# Crystal structure of 2-pentyl­oxybenzamide

**DOI:** 10.1107/S1600536814020571

**Published:** 2014-09-20

**Authors:** Bernhard Bugenhagen, Yosef Al Jasem, Thies Thiemann

**Affiliations:** a Institute of Inorganic Chemistry, University of Hamburg, Hamburg, Germany; b Department of Chemical Engineering, United Arab Emirates University, AL Ain, Abu Dhabi, United Arab Emirates; c Department of Chemistry, United Arab Emirates University, AL Ain, Abu Dhabi, United Arab Emirates

**Keywords:** crystal structure, 2-alk­oxy­benzamide, hydrogen bonding.

## Abstract

In the title mol­ecule, C_12_H_17_NO_2_, the amide NH_2_ group is oriented toward the pent­yloxy substituent and an intra­molecular N—H⋯O hydrogen bond is formed with the pent­yloxy O atom. The benzene ring forms dihedral angles of 2.93 (2) and 5.60 (2)° with the amide group and the pent­yloxy group mean planes, respectively. In the crystal, mol­ecules are linked by pairs of N—H⋯O hydrogen bonds, forming inversion dimers with their mol­ecular planes parallel, but at an offset of 0.45 (1) Å to each other. These dimers are ordered into two types of symmetry-related columns extended along the *a* axis, with the mean plane of one set of dimers in a column approximately parallel to (121) and the other in a column approximately parallel to (1-21). The two planes form a dihedral angle of 85.31 (2)°, and are linked *via* C—H⋯O hydrogen bonds and C—H⋯π inter­actions, forming a three-dimensional framework structure.

## Abstract 2

In the nearly planar 2-pentyl­oxybenzamide mol­ecule, there is an intra­molecular N—H⋯O hydrogen bond involving one amide proton and the ether oxygen. In the crystal, pairs of N—H⋯O hydrogen bonds organize mol­ecules into inversion dimers lying in two planes, (121) and (1
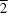
1).

## Chemical context   

2-Alk­oxy­benzamide moieties can be found as structural units in medicinally active compounds, such in dopamine (DA) receptor antagonists (van de Waterbeemd & Testa, 1983 ▶). Typically such components are Sulpiride^®^, Metoclopramide^®^ and Tiapride^®^. Other substituted 2-alk­oxy­benzamides have been found to be antagonists of chemotherapy-induced nausea (Monkovic *et al.*, 1988 ▶). Also, 2-alk­oxy­benzamides have been proposed as agonists of the α7 nicotinic receptor (Bodnar *et al.*, 2005 ▶) and as neuroleptic compounds (Florvall & Oegren, 1982 ▶). 2-Eth­oxy­benzamide, under the name ethenzamide, is a commonly used analgesicum (Darias *et al.*, 1992 ▶). 
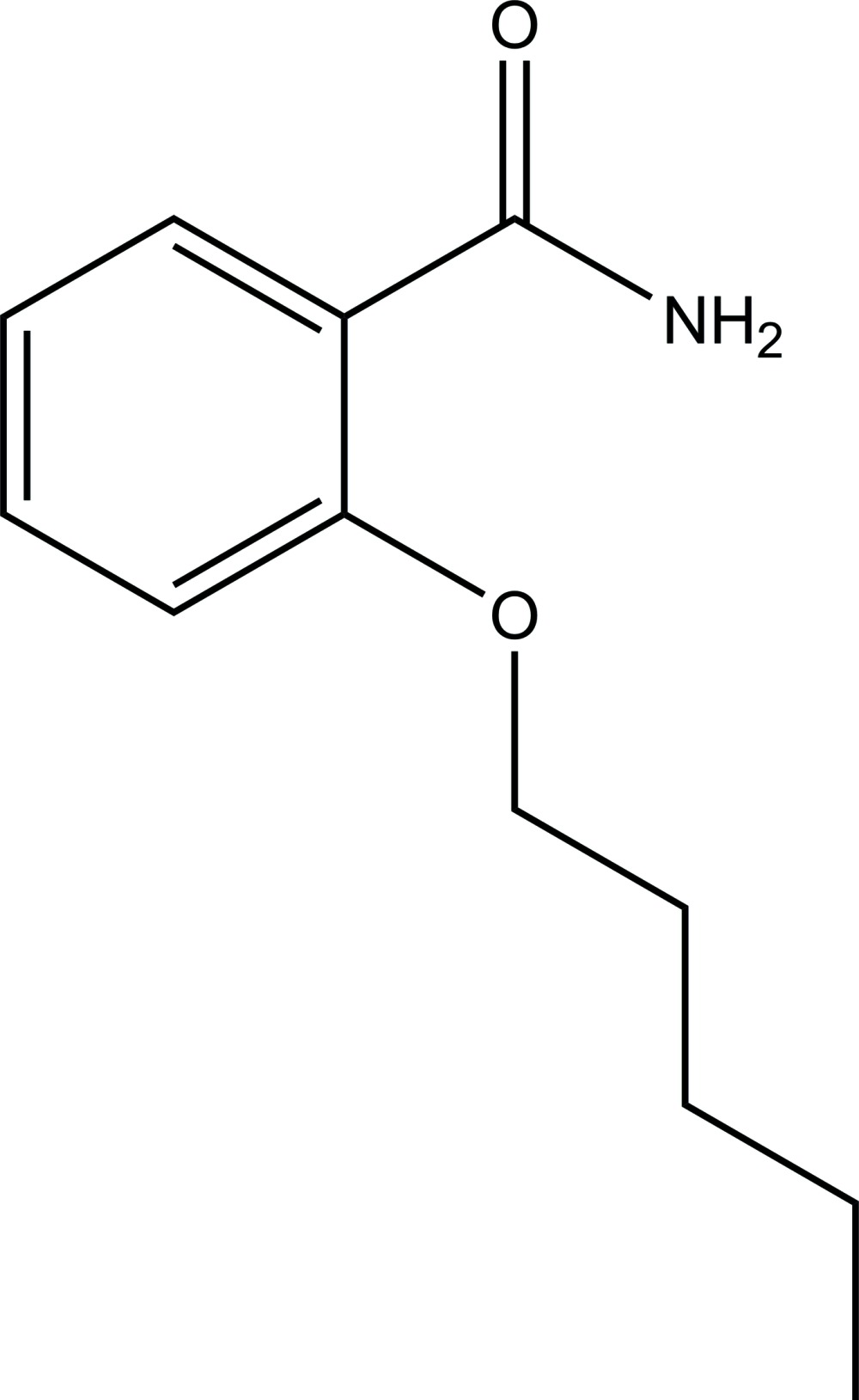


In our efforts to use 2-alk­oxy­benzamides as components in co-crystal formation (Aitipamula *et al.*, 2012 ▶), we prepared the title compound, 2-pentyl­oxybenzamide, and report herein on its crystal structure. 2-Pentyl­oxybenzamide was first studied for its anti­pyretic and analgesic properties (Bavin *et al.*, 1952 ▶; Macrae & Seymour, 1956 ▶). Afterwards, it was found to have anti­fungal activity and to be useful in the treatment of dermatomycosis (Simmonite & Tattersall, 1962 ▶; Coates *et al.*, 1957 ▶). Under the name penta­lamide, it is still used as an ingredient in anti­fungal agents for topical use.

## Structural commentary   

**Figure 1:**
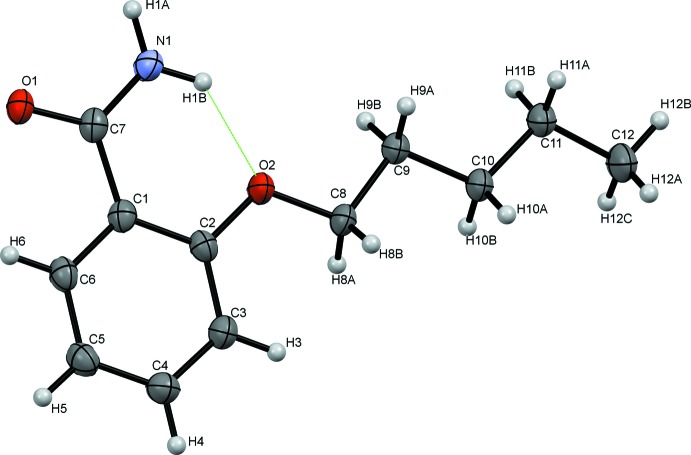
A view of the mol­ecular structure of the title mol­ecule, with atom labelling. Displacement ellipsoids are shown at the 50% probability level. The intra­molecular N—H⋯O hydrogen bond is shown as a green dashed line (see Table 1 ▶ for details).

In the title mol­ecule, Fig. 1 ▶, the benzene ring is nearly coplanar with the amide group [C6—C1—C7—O1 = −2.48 (18)°] and the pent­yloxy group [C3—C2—O2—C8 = 0.37 (18)°]. The amide NH_2_ group is oriented towards the ether group allowing for an intra­molecular hydrogen bond (N1—H1*B*⋯O2; Fig. 1 ▶ and Table 1 ▶). The latter is also present in analogous compounds, such as 3-hy­droxy-2-meth­oxy­benzamide (Wilbrand *et al.*, 2012 ▶), 2-propoxybenzamide (Al Jasem *et al.*, 2012 ▶) and 2-(prop-2-en­yloxy)benzamide (Bugenhagen *et al.*, 2012 ▶). 2-Eth­oxy­benzamide is the only studied 2-alk­oxy­benzamide that does not exhibit an intra­molecular hydrogen bond in the single component crystal (Pagola & Stephens, 2009 ▶). However, it shows a similar conformation to the other 2-alk­oxy­benzamides in the co-crystal form with thio­urea (Moribe *et al.*, 2004 ▶), and with salicylic acid (Back *et al.*, 2012 ▶).

## Supra­molecular features   

**Figure 2:**
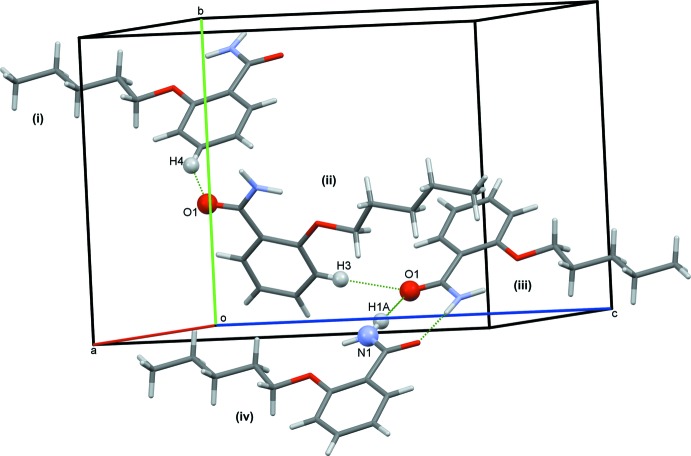
A partial view of the crystal packing of the title compound. The hydrogen bonds are shown as green dashed lines [see Table 1 ▶ for details; symmetry codes: (i) −*x* + 1, *y* + 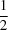, −*z* + 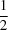; (ii) *x*, *y*, *z*; (iii) *x*, −*y* + 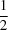, *z* + 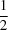; (iv) −*x*, *y* − 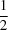, −*z* + 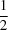]

**Figure 3:**
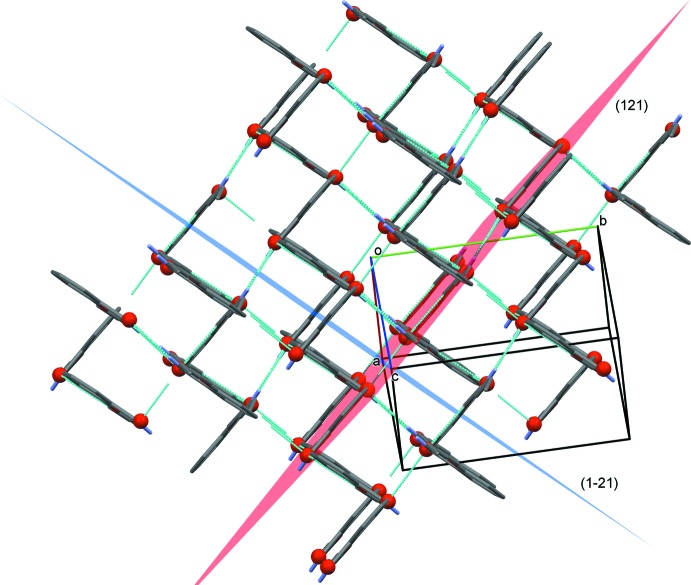
A view of the crystal network formed by the layers of inversion dimers in the planes (121) in red, and (1
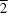
1) in blue. The hydrogen bonds are shown as green dashed lines (see Table 1 ▶ for details; H atoms have been omitted for clarity).

In the crystal, mol­ecules are linked by pairs of N-H⋯O (N1—H1*A*⋯O1) hydrogen bonds forming inversion dimers (Fig. 2 ▶ and Table 1 ▶). These dimers form a nested network of mol­ecules, made of two layers, (121) and (1
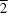
1), which form an angle of 85.31 (2)° between their planes (Fig. 3 ▶). The dimers in the layers are linked by C—H⋯O (C4—H4⋯O1) hydrogen bonds and C—H⋯π inter­actions, forming a three-dimensional framework (Fig. 3 ▶ and Table 1 ▶). Within two parallel layers, pairs of mol­ecules lie with an offset to each other without any noticeable, direct inter­action between them; the parallel layers are at a distance of 3.81 (3) Å from each other. Along the *a* axis the pairs are ordered in two symmetry-related columns. The plane of the benzene ring (C1–C6) of the 2-pentyl­oxybenzamide forms an angle of 25.29 (2)° with the column axis.

## Database survey   

From a database survey (Cambridge Structural Database, Version 5.35, last update May 2014; Allen, 2002 ▶), the following were picked as relevant comparable structures: 3-hy­droxy-2-meth­oxy­benzamide (Wilbrand *et al.*, 2012 ▶), 2-meth­oxy­benzamide (Moribe *et al.*, 2006 ▶), 2-eth­oxy­benzamide (Pagola & Stephens, 2009 ▶; Back *et al.*, 2012 ▶), 2-propoxybenzamide (Al Jasem *et al.*, 2012 ▶) and 2-(prop-2-en­yloxy)benzamide (Bugen­hagen *et al.*, 2012 ▶). For 2-propoxybenzamide, a homologue of the title compound, a similar formation of inversion-related mol­ecular pairs in the crystal was reported, hence the two compounds exhibit a similar packing. The noticeable difference between the two compounds is the larger dihedral angle between the carboxamide group and the benzene ring in 2-propoxybenzamide, 12.41 (2)° compared to 3.30 (15)° in the title compound, 2-pentyl­oxybenzamide. Also, the parallel layers of mol­ecules in the title compound are further apart [separated by 3.81 (3) Å] than is found for a similar packing of 2-propoxybenzamide [3.69 (2) Å]. Similarly, inversion-related pairs of mol­ecules are formed by inter­molecular (amide–amide) hydrogen bonding in 2-eth­oxy­benzamide and 3-hy­droxy-2-meth­oxy­benzamide. As 2-eth­oxy­benzamide exhibits no intra­molecular hydrogen bonding, the freed acceptor–donor sites are used for additional inter­molecular hydrogen bonding with the adjacent mol­ecule.

In contrast, in 2-meth­oxy­benzamide and in 2-(prop-2-en­yloxy)benzamide the inter­molecular N—H⋯O hydrogen bonds involving the amide groups do not lead to pair formation but generate *C*(4) and 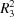
(7) motifs.

## Synthesis and crystallization   

The preparation of the title compound follows a Williamson ether synthesis using DMSO as solvent, analogous to a general procedure (Johnstone & Rose, 1979 ▶): To powdered KOH (1.12 g, 20.0 mmol) in DMSO (18 ml) was added salicyl­amide (1.37 g, 10.0 mmol), and the resulting mixture was stirred for 10 min. at rt. Then, *n*-amyl iodide (4.2 g, mmol, 21.2 mmol) was added dropwise. The solution was stirred for 12 h at rt. It was then poured into water (200 ml) and extracted with chloro­form (3 × 75 ml). The organic phase was dried over anhydrous MgSO_4_, concentrated *in vacuo*, and the residue was subjected to column chromatography on silica gel (CHCl_3_/M^*t*^BE/hexane *v/v/v* 1:1:1) to give the title compound (1.55 g, 75%) as colourless crystals (m.p. 362 K). IR (KBr, cm^−1^) *ν*max 3434, 3168, 2948, 2868, 1664, 1593, 1387, 1232, 1164, 1018, 832, 788, 765, 575; ^1^H NMR (400 MHz, CDCl_3_, *δ*_H_) 0.93 (3H, *t*, ^3^ *J* = 7.2 Hz, CH_3_), 1.38–1.48 (4H, *m*), 1.84–1.89 (2H, *m*), 4.11 (2*H*, *d*, ^3^ *J* = 6.4 Hz); 6.03 (1H, *bs*, NH), 6.96 (1H, *d*, ^3^ *J* = 8.4 Hz), 7.03–7.07 (1H, *m*), 7.42–7.46 (1H, *m*), 7.85 (1H, *bs*, NH), 8.20 (1H, *dd*, ^3^ *J* = 7.6 Hz, ^4^ *J* = 2.0 Hz), ^13^C NMR (100.5 MHz, CDCl_3_, *δ*_C_) 14.0 (CH_3_), 22.4 (CH_2_), 28.2 (CH_2_), 28.9 (CH_2_), 69.1 (OCH_2_), 112.2 (CH), 120.7 (C_quat_), 121.0 (CH), 132.5 (CH), 133.3 (C_quat_), 157.4 (C_quat_), 167.2 (C_quat_, CO).

## Refinement   

Crystal data, data collection and structure refinement details are summarized in Table 2 ▶. All C-bound H atoms were placed in calculated positions and refined as riding atoms: C—H distances of 0.95 − 1.00 Å with *U*_iso_(H) = *xU*_eq_(C), where *x* = 1.5 for methyl and = 1.2 for other H-atoms. The N-bound H atoms were located in a difference electron-density map and freely refined.

## Supplementary Material

Crystal structure: contains datablock(s) I. DOI: 10.1107/S1600536814020571/su2779sup1.cif

Structure factors: contains datablock(s) I. DOI: 10.1107/S1600536814020571/su2779Isup2.hkl

Supporting information file. DOI: 10.1107/S1600536814020571/su2779Isup3.cml

CCDC reference: 1024316

Additional supporting information:  crystallographic information; 3D view; checkCIF report

## Figures and Tables

**Table 1:** Hydrogen-bond geometry (Å, °) *Cg*1 is the centroid of the C1–C6 benzene ring.

*D*—H⋯*A*	*D*—H	H⋯*A*	*D*⋯*A*	*D*—H⋯*A*
N1—H1*B*⋯O2	0.915 (17)	1.921 (18)	2.6510 (15)	135.4 (15)
N1—H1*A*⋯O1^i^	0.919 (19)	1.964 (19)	2.8824 (15)	177.8 (17)
C3—H3⋯O1^ii^	0.93	2.62	3.546 (2)	178
C4—H4⋯O1^iii^	0.93	2.53	3.306 (2)	141
C11—H11*A*⋯*Cg*1^iv^	0.97	2.90	3.7283 (16)	141

Symmetry codes: (i) 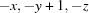; (ii) 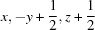; (iii) 
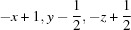
; (iv) 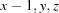.

**Table 2:** Experimental details

Crystal data	
Chemical formula	C_12_H_17_NO_2_
*M*_r_	207.27
Crystal system, space group	Monoclinic, *P*2_1_/*c*
Temperature (K)	100
*a*, *b*, *c* (Å)	8.1830 (2), 11.2706 (2), 14.5386 (4)
β (°)	119.696 (2)
*V* (Å^3^)	1164.76 (5)
*Z*	4
Radiation type	Cu *K*α
μ (mm^−1^)	0.64
Crystal size (mm)	0.25 × 0.19 × 0.10

Data collection	
Diffractometer	SuperNova, Dual, Cu at zero, Atlas
Absorption correction	Multi-scan (*CrysAlis PRO*; Agilent, 2012 ▶)
*T*_min_, *T*_max_	0.854, 1.000
No. of measured, independent and observed [*I* > 2σ(*I*)] reflections	6112, 2268, 1900
*R*_int_	0.022
(sin θ/λ)_max_ (Å^−1^)	0.621

Refinement	
*R*[*F* ^2^ > 2σ(*F* ^2^)], *wR*(*F* ^2^), *S*	0.041, 0.115, 1.03
No. of reflections	2268
No. of parameters	145
H-atom treatment	H atoms treated by a mixture of independent and constrained refinement
Δρ_max_, Δρ_min_ (e Å^−3^)	0.19, −0.22

Computer programs: *CrysAlis PRO* (Agilent, 2012 ▶), *SHELXS97* and *SHELXL97* (Sheldrick, 2008 ▶) within *OLEX2* (Dolomanov *et al.*, 2009 ▶), *PLATON* (Spek, 2009 ▶) and *Mercury* (Macrae *et al.*, 2008 ▶).

## supplementary crystallographic information

### Crystal data

**Table:**

C_12_H_17_NO_2_	*F*(000) = 448
*M**_r_* = 207.27	*D*_x_ = 1.182 Mg m^−^^3^
Monoclinic, *P*2_1_/*c*	Cu *K*α radiation, λ = 1.5418 Å
*a* = 8.1830 (2) Å	Cell parameters from 2743 reflections
*b* = 11.2706 (2) Å	θ = 3.9–73.1°
*c* = 14.5386 (4) Å	µ = 0.64 mm^−^^1^
β = 119.696 (2)°	*T* = 100 K
*V* = 1164.76 (5) Å^3^	Block, colourless
*Z* = 4	0.25 × 0.19 × 0.10 mm

### Data collection

**Table:**

SuperNova, Dual, Cu at zero, Atlas diffractometer	2268 independent reflections
Radiation source: SuperNova (Cu) X-ray Source	1900 reflections with *I* > 2σ(*I*)
Mirror monochromator	*R*_int_ = 0.022
Detector resolution: 10.4127 pixels mm^-1^	θ_max_ = 73.3°, θ_min_ = 5.3°
ω scans	*h* = −10→5
Absorption correction: multi-scan (*CrysAlis PRO*; Agilent, 2012)	*k* = −13→14
*T*_min_ = 0.854, *T*_max_ = 1.000	*l* = −16→18
6112 measured reflections	

### Refinement

**Table:**

Refinement on *F*^2^	Primary atom site location: structure-invariant direct methods
Least-squares matrix: full	Secondary atom site location: difference Fourier map
*R*[*F*^2^ > 2σ(*F*^2^)] = 0.041	Hydrogen site location: inferred from neighbouring sites
*wR*(*F*^2^) = 0.115	H atoms treated by a mixture of independent and constrained refinement
*S* = 1.03	*w* = 1/[σ^2^(*F*_o_^2^) + (0.0645*P*)^2^ + 0.2593*P*] where *P* = (*F*_o_^2^ + 2*F*_c_^2^)/3
2268 reflections	(Δ/σ)_max_ < 0.001
145 parameters	Δρ_max_ = 0.19 e Å^−^^3^
0 restraints	Δρ_min_ = −0.22 e Å^−^^3^

### Special details

**Table:**

Geometry. All e.s.d.'s (except the e.s.d. in the dihedral angle between two l.s. planes) are estimated using the full covariance matrix. The cell e.s.d.'s are taken into account individually in the estimation of e.s.d.'s in distances, angles and torsion angles; correlations between e.s.d.'s in cell parameters are only used when they are defined by crystal symmetry. An approximate (isotropic) treatment of cell e.s.d.'s is used for estimating e.s.d.'s involving l.s. planes.
Refinement. Refinement of *F*^2^ against ALL reflections. The weighted *R*-factor *wR* and goodness of fit *S* are based on *F*^2^, conventional *R*-factors *R* are based on *F*, with *F* set to zero for negative *F*^2^. The threshold expression of *F*^2^ > σ(*F*^2^) is used only for calculating *R*-factors(gt) *etc*. and is not relevant to the choice of reflections for refinement. *R*-factors based on *F*^2^ are statistically about twice as large as those based on *F*, and *R*- factors based on ALL data will be even larger.

### Fractional atomic coordinates and isotropic or equivalent isotropic displacement parameters (Å^2^)

**Table:**

	*x*	*y*	*z*	*U*_iso_*/*U*_eq_	
C1	0.25614 (18)	0.29076 (11)	0.20599 (10)	0.0233 (3)	
C10	−0.14119 (19)	0.33945 (13)	0.46481 (11)	0.0269 (3)	
C11	−0.29380 (19)	0.41079 (13)	0.47067 (11)	0.0294 (3)	
C12	−0.3051 (2)	0.37915 (16)	0.56955 (12)	0.0389 (4)	
C2	0.21475 (18)	0.25854 (12)	0.28594 (10)	0.0240 (3)	
C3	0.31443 (19)	0.16670 (12)	0.35597 (11)	0.0279 (3)	
C4	0.45350 (19)	0.10654 (13)	0.34698 (11)	0.0303 (3)	
C5	0.49609 (19)	0.13709 (13)	0.26885 (11)	0.0301 (3)	
C6	0.39777 (18)	0.22849 (12)	0.19968 (10)	0.0266 (3)	
C7	0.15680 (18)	0.38484 (11)	0.12366 (10)	0.0232 (3)	
C8	0.03619 (19)	0.28821 (12)	0.37434 (10)	0.0263 (3)	
C9	−0.11424 (18)	0.36885 (12)	0.37063 (10)	0.0256 (3)	
H10A	−0.1714	0.2558	0.4618	0.032*	
H10B	−0.0229	0.3526	0.5295	0.032*	
H11A	−0.4142	0.3950	0.4082	0.035*	
H11B	−0.2672	0.4948	0.4717	0.035*	
H12A	−0.3329	0.2962	0.5683	0.058*	
H12B	−0.4027	0.4250	0.5710	0.058*	
H12C	−0.1869	0.3963	0.6315	0.058*	
H1A	−0.056 (2)	0.4934 (17)	0.0658 (14)	0.042 (5)*	
H1B	−0.021 (2)	0.4242 (16)	0.1713 (14)	0.041 (5)*	
H3	0.2873	0.1458	0.4089	0.033*	
H4	0.5188	0.0452	0.3937	0.036*	
H5	0.5896	0.0967	0.2630	0.036*	
H6	0.4270	0.2490	0.1475	0.032*	
H8A	0.1495	0.2950	0.4427	0.032*	
H8B	−0.0062	0.2065	0.3650	0.032*	
H9A	−0.2313	0.3570	0.3049	0.031*	
H9B	−0.0767	0.4511	0.3742	0.031*	
N1	0.01348 (16)	0.44361 (11)	0.12198 (9)	0.0282 (3)	
O1	0.20767 (12)	0.40558 (9)	0.05777 (7)	0.0264 (2)	
O2	0.07616 (13)	0.32058 (8)	0.29182 (7)	0.0271 (2)	

### Atomic displacement parameters (Å^2^)

**Table:**

	*U*^11^	*U*^22^	*U*^33^	*U*^12^	*U*^13^	*U*^23^
O1	0.0293 (5)	0.0312 (5)	0.0239 (5)	−0.0023 (4)	0.0171 (4)	0.0000 (4)
O2	0.0312 (5)	0.0313 (5)	0.0271 (5)	0.0063 (4)	0.0207 (4)	0.0059 (4)
N1	0.0343 (6)	0.0299 (6)	0.0271 (6)	0.0063 (5)	0.0204 (5)	0.0060 (5)
C1	0.0233 (6)	0.0263 (6)	0.0213 (6)	−0.0034 (5)	0.0116 (5)	−0.0028 (5)
C2	0.0227 (6)	0.0269 (7)	0.0250 (6)	−0.0002 (5)	0.0139 (5)	−0.0021 (5)
C3	0.0293 (7)	0.0316 (7)	0.0266 (7)	0.0015 (5)	0.0168 (6)	0.0028 (5)
C4	0.0302 (7)	0.0313 (7)	0.0300 (7)	0.0060 (6)	0.0153 (6)	0.0045 (6)
C5	0.0279 (7)	0.0347 (8)	0.0309 (7)	0.0044 (6)	0.0169 (6)	−0.0018 (6)
C6	0.0259 (7)	0.0331 (7)	0.0244 (6)	−0.0013 (5)	0.0153 (6)	−0.0028 (5)
C7	0.0252 (7)	0.0244 (6)	0.0224 (6)	−0.0053 (5)	0.0135 (5)	−0.0046 (5)
C8	0.0303 (7)	0.0300 (7)	0.0250 (6)	0.0035 (5)	0.0186 (6)	0.0055 (5)
C9	0.0260 (7)	0.0296 (7)	0.0247 (6)	0.0022 (5)	0.0152 (5)	0.0034 (5)
C10	0.0281 (7)	0.0318 (7)	0.0259 (7)	0.0031 (6)	0.0173 (6)	0.0039 (5)
C11	0.0290 (7)	0.0357 (7)	0.0284 (7)	0.0037 (6)	0.0180 (6)	0.0032 (6)
C12	0.0405 (9)	0.0513 (10)	0.0375 (8)	0.0100 (7)	0.0288 (7)	0.0064 (7)

### Geometric parameters (Å, º)

**Table:**

O1—C7	1.2412 (15)	C6—H6	0.9300
O2—C2	1.3706 (15)	C8—H8A	0.9700
O2—C8	1.4381 (14)	C8—H8B	0.9700
N1—C7	1.3366 (17)	C8—C9	1.5095 (18)
N1—H1A	0.919 (19)	C9—H9A	0.9700
N1—H1B	0.915 (17)	C9—H9B	0.9700
C1—C2	1.4099 (17)	C9—C10	1.5272 (17)
C1—C6	1.3962 (18)	C10—H10A	0.9700
C1—C7	1.5013 (18)	C10—H10B	0.9700
C2—C3	1.3966 (19)	C10—C11	1.5227 (18)
C3—H3	0.9300	C11—H11A	0.9700
C3—C4	1.3853 (19)	C11—H11B	0.9700
C4—H4	0.9300	C11—C12	1.5278 (18)
C4—C5	1.3866 (19)	C12—H12A	0.9600
C5—H5	0.9300	C12—H12B	0.9600
C5—C6	1.385 (2)	C12—H12C	0.9600
			
C1—C6—H6	119.0	C6—C1—C7	116.23 (11)
C10—C11—C12	111.28 (12)	C6—C1—C2	118.09 (12)
C10—C11—H11B	109.4	C7—N1—H1B	118.2 (11)
C10—C11—H11A	109.4	C7—N1—H1A	118.0 (10)
C10—C9—H9B	110.0	C8—C9—C10	108.35 (11)
C10—C9—H9A	110.0	C8—C9—H9B	110.0
C11—C12—H12C	109.5	C8—C9—H9A	110.0
C11—C12—H12B	109.5	C9—C10—H10B	108.5
C11—C12—H12A	109.5	C9—C10—H10A	108.5
C11—C10—H10B	108.5	C9—C8—H8B	109.7
C11—C10—H10A	108.5	C9—C8—H8A	109.7
C11—C10—C9	114.98 (11)	H10A—C10—H10B	107.5
C12—C11—H11B	109.4	H11A—C11—H11B	108.0
C12—C11—H11A	109.4	H12A—C12—H12C	109.5
C2—C3—H3	119.9	H12A—C12—H12B	109.5
C2—C1—C7	125.65 (12)	H12B—C12—H12C	109.5
C2—O2—C8	117.38 (10)	H1A—N1—H1B	123.2 (14)
C3—C4—C5	120.48 (13)	H8A—C8—H8B	108.2
C3—C4—H4	119.8	H9A—C9—H9B	108.4
C3—C2—C1	119.99 (12)	N1—C7—C1	119.22 (11)
C4—C5—H5	120.4	O1—C7—C1	119.48 (11)
C4—C3—H3	119.9	O1—C7—N1	121.29 (12)
C4—C3—C2	120.29 (12)	O2—C8—C9	109.63 (10)
C5—C6—H6	119.0	O2—C8—H8B	109.7
C5—C6—C1	121.92 (12)	O2—C8—H8A	109.7
C5—C4—H4	119.8	O2—C2—C3	122.35 (11)
C6—C5—H5	120.4	O2—C2—C1	117.66 (11)
C6—C5—C4	119.22 (12)		
			
C1—C2—C3—C4	−0.3 (2)	C6—C1—C2—O2	179.67 (11)
C2—C3—C4—C5	0.3 (2)	C7—C1—C6—C5	−177.98 (12)
C2—C1—C7—N1	−1.4 (2)	C7—C1—C2—C3	178.07 (12)
C2—C1—C7—O1	179.44 (12)	C7—C1—C2—O2	−2.27 (19)
C2—C1—C6—C5	0.3 (2)	C8—C9—C10—C11	−178.10 (12)
C2—O2—C8—C9	177.89 (11)	C8—O2—C2—C3	0.37 (18)
C3—C4—C5—C6	−0.1 (2)	C8—O2—C2—C1	−179.28 (11)
C4—C5—C6—C1	−0.2 (2)	C9—C10—C11—C12	−177.64 (12)
C6—C1—C7—N1	176.66 (12)	O2—C8—C9—C10	−175.11 (10)
C6—C1—C7—O1	−2.48 (18)	O2—C2—C3—C4	−179.95 (12)
C6—C1—C2—C3	0.02 (19)		

### Hydrogen-bond geometry (Å, º)

Cg1 is the centroid of the C1–C6 benzene ring.

**Table:**

*D*—H···*A*	*D*—H	H···*A*	*D*···*A*	*D*—H···*A*
N1—H1*B*···O2	0.915 (17)	1.921 (18)	2.6510 (15)	135.4 (15)
N1—H1*A*···O1^i^	0.919 (19)	1.964 (19)	2.8824 (15)	177.8 (17)
C3—H3···O1^ii^	0.93	2.62	3.546 (2)	178
C4—H4···O1^iii^	0.93	2.53	3.306 (2)	141
C11—H11*A*···*Cg*1^iv^	0.97	2.90	3.7283 (16)	141

Symmetry codes: (i) −*x*, −*y*+1, −*z*; (ii) *x*, −*y*+1/2, *z*+1/2; (iii) −*x*+1, *y*−1/2, −*z*+1/2; (iv) *x*−1, *y*, *z*.
